# A Novel Spine Fixation System Made Entirely of Carbon-Fiber-Reinforced PEEK Composite: An In Vitro Mechanical Evaluation

**DOI:** 10.1155/2020/4796136

**Published:** 2020-06-09

**Authors:** Ofir Uri, Yoram Folman, Gil Laufer, Eyal Behrbalk

**Affiliations:** Department of Orthopedic Surgery, Hillel Yaffe Medical Center, Hadera, Israel

## Abstract

**Background:**

Semirigid spine fixation systems utilizing nonmetallic materials have emerged as a promising innovation to overcome the inherent disadvantages of metal instrumentation in spine surgery. This study tests the mechanical properties of a novel spine fixation system made entirely of carbon-fiber-reinforced PEEK (CFR-PEEK) composite material (CarboClear System, CarboFix Orthopedics Ltd., Israel).

**Methods:**

An in vitro mechanical evaluation of the CFR-PEEK CarboClear system was conducted in compliance with the American Society for Testing and Materials (ASTM) F1717, F2193, and F543 standards.

**Results:**

The mean bending yield load, bending ultimate load, and bending stiffness of the construct were 322 N, 363 N, and 45 N/mm, respectively. All tested samples completed 5 × 10^6^ dynamic cycles successfully, with no evidence of fatigue failure at increasing load levels, up to 83% of ultimate bending load. The mean torsional stiffness was 1.0 Nm/deg and the mean screw axial pull-out strength was 2,037 N.

**Conclusion:**

The CarboClear Pedicle Screw System has mechanical properties comparable to those of other commonly used titanium-made systems, with superior fatigue properties. The fatigue resistance, modulus of elasticity which is very similar to that of bone, radiolucency, and CT/MRI artifact-free feature of this spine fixation system made entirely of CFR-PEEK may offer advantages over traditional spine fixation systems made of metal alloys.

## 1. Introduction

The use of rigid metal instrumentation in spine surgery has become common practice over recent decades [1]. Although such instrumentation has dramatically increased the union rate in spinal fusion [2, 3], evidence suggests that the stiffness of the metallic implants used far exceeds the requirements for successful fusion and may lead to postoperative complications such as adjacent level degeneration [4, 5], vertebral body osteopenia related to the load bearing effect of rigid instrumentation [6], and screw loosening, especially in osteoporotic patients [7]. Moreover, metal-induced scattering associated with the use of metal alloys in spinal fusion reduces the reliability of imaging modalities such as CT, MRI in the postoperative follow-up, and treatment [8].

Semirigid fixation systems that utilize nonmetallic materials were developed to address these inherent disadvantages of metal instrumentation [9]. One of the material alternatives is the thermoplastic polyetheretherketone (PEEK) biocompatible polymer. Biomechanical studies comparing rods of posterior spinal fusion systems made of Ti-alloy versus PEEK demonstrated that the PEEK-rod system provides increased load sharing along the anterior column and lower stress at the bone-screw interface. This potentially reduces the risk of adjacent level degeneration, vertebral body bone loss, and screw loosening [9, 10]. Being radiographically translucent, spinal instrumentation with rods made of PEEK interferes less with postoperative imaging, thus enabling easier radiographic follow-up at the rod area [9, 10].

A remaining disadvantage of the semirigid PEEK-rod systems is its inability to provide sufficient primary stability to allow spinal fusion due to micromovements attributed to the lower strength and modulus of elasticity of the PEEK compared to metals [8–10]. In order to provide PEEK with more strength and stiffness, a composite of PEEK-based matrix reinforced by carbon fibers (CFR-PEEK) has been introduced in orthopedic and spine surgery over the past years. It has been shown that orthopedic implants made of CFR-PEEK composite, with carbon fibers fraction of approximately 60% by volume, have mechanical properties (e.g., modulus of elasticity and fatigue strain) equivalent to that of cortical bone with promising results in spine surgery [11–14].

The CarboClear Pedicle Screw System (CarboFix Orthopedics Ltd., Herzliya, Israel) is a novel spine fixation system composed entirely of CFR-PEEK composite (i.e., rods and pedicle screws).

Preliminary reports of the CarboClear system for the treatment of spine tumors were recently published and showed outcomes comparable to the standard titanium system in terms of complications, stability, and functional scores [13, 14].

**Figure 1:**
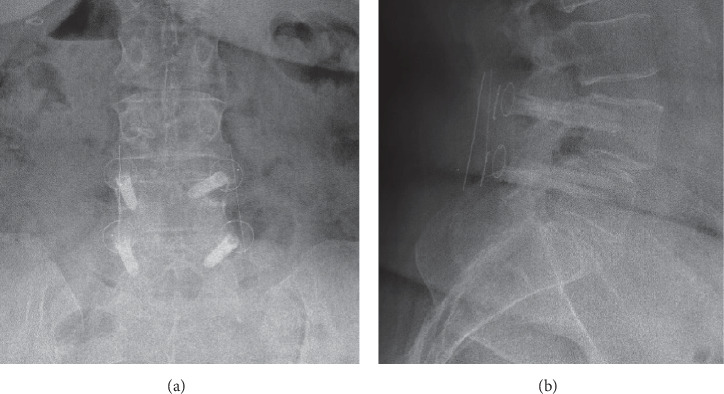
The CarboClear spine fixation system. (a) Anterior-posterior and (b) lateral lumbar spine radiographs of a 64-year-old female at 3-year follow-up after spinal fusion with the radiolucent carbon-fiber-reinforced PEEK composite CarboClear system.

This study investigates the mechanical properties of the CarboClear system. To our knowledge, no previous study has evaluated the mechanical properties of the spine fixation system made entirely of CFR-PEEK composite. (Figure 1).

## 2. Materials and Methods

The study was conducted at the manufacturer's laboratories, CarboFix Orthopedics Ltd. (Herzliya, Israel), who provided financial support for the study, but was not involved in the manuscript writing and editing. All experiments were conducted under the strict protocols of the American Society for Testing and Materials (ASTM).

The CFR-PEEK specimens tested comprised 6.5 mm polyaxial pedicle screws with 6.0 mm straight rods and their locking elements. The manufacturer instructions were strictly kept throughout implant assembly.

**Figure 2:**
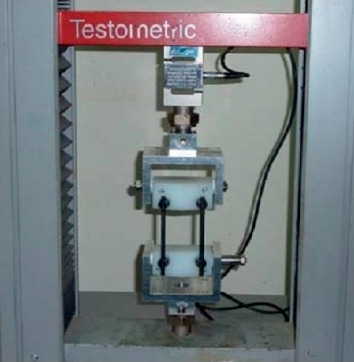
Static axial compression bending test performed in compliance with the ASTM F1717 standard, using a Testometric M350-10 kN testing machine, and simulating a vertebrectomy model via a large gap between two UHMWPE blocks simulating two vertebrae.

**Figure 3:**
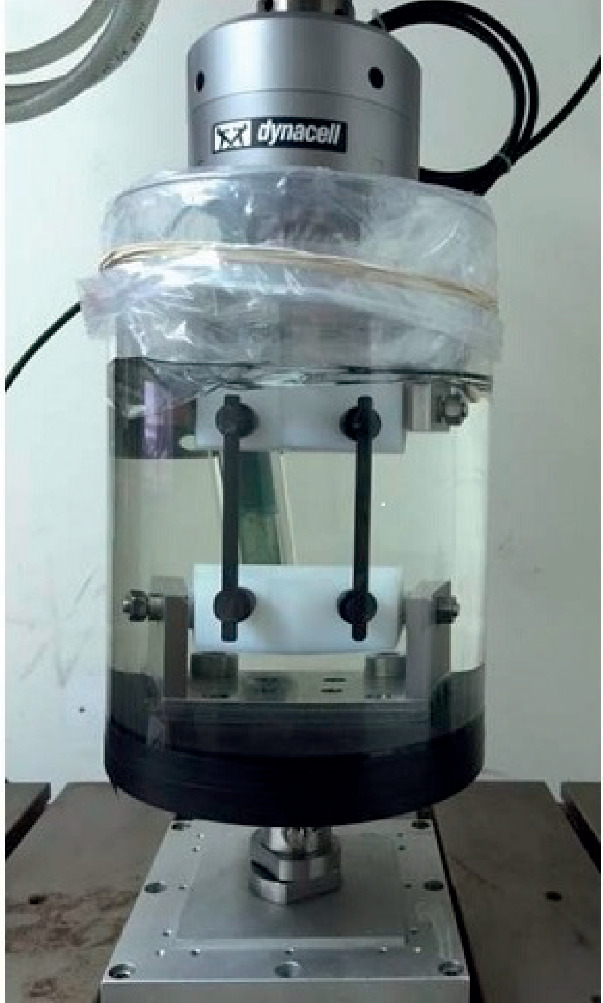
Fatigue axial compression bending test performed in compliance with the ASTM F1717 standard, using an Instron 8871 fatigue system testing machine, in phosphate-buffered saline solution setup at 37°C.

**Figure 4:**
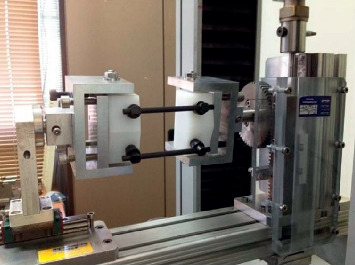
Static torsion test performed in compliance with the ASTM F1717 standard using a Testometric M350-10 kN testing machine at a rate of 6 deg/min.

**Figure 5:**
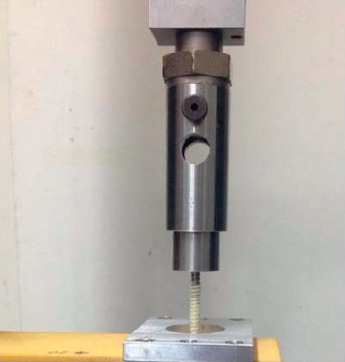
Screw axial pull-out strength test performed in compliance with ASTM F 2193 and ASTM F 543 standards using a Testometric M350-10 kN testing machine.

The following mechanical tests were performed:

1. Static axial compression bending test: the test was performed according to the ASTM F1717 standard [15], simulating a vertebrectomy model via a large gap between two ultrahigh-molecular weight polyethylene (UHMWPE) blocks simulating two vertebrae. Six CFR-PEEK constructs were tested, each comprising four pedicle screws and two rods. The apparatus included two UHMWPE blocks (with tensile breaking strength of 40 ± 3 MPa), mounted by metal hinge pins to side supports on the test machine (Testometric M350-10 kN, Testometric Ltd., UK) and attached to the actuator and to the load cell. The upper side support-upper UHMWPE block construct and the lower side support-UHMWPE block construct were aligned. The center axis of each hinge pin was perpendicular to and aligned with the load axis of the test machine. As per ASTM F1717 recommendation, the active length of the longitudinal element (rod) was set at 76 mm and the block moment arm at 40 mm (Figure 2). Loading rate was 5 mm/min. Test plots of load versus displacement were generated for each assembly tested and the mode of failure of each construct was documented.
2. Fatigue axial compression bending test: the test was performed according to the ASTM F1717 protocol. Six CFR-PEEK constructs were tested, each comprising four pedicle screws and two rods. A similar apparatus as for the static axial compression bending test was utilized. The samples were tested using an Instron 8871 fatigue system. The samples were evaluated at a number of increased load levels (defined as a percentage of the average ultimate axial compression bending load found at the static axial compression bending test described above, i.e., 50%, 75%). The constructs were tested in phosphate-buffered saline solution at a temperature of 37°C. The cycle rate was set at 2 Hz. The endpoint of the test was defined as construct failure or completion of 5 × 10^6^ cycles without failure. Plots of load vs. number of cycles and displacement vs. number of cycles were generated (Figure 3).
3. Static torsion test: again, the test was performed according to the ASTM F1717 protocol, using a similar apparatus with the addition of aluminum blocks between the UHMWPE blocks and the base plate to stop rotation around the hinge pin. Four CFR-PEEK constructs were tested, each comprising four pedicle screws and two rods. The samples were tested using a tension/compression testing machine (Testometric M350-10 kN) at a rate of 6 deg/min. Plots of torque vs. angular displacement were generated for each assembly tested (Figure 4).
4. Screw axial pull-out strength test: this test was designed to measure the axial pull-out strength of the CFR-PEEK pedicle screw and was performed according to ASTM F2193 and ASTM F543 standards [15, 16]. Four CFR-PEEK polyaxial pedicle screws (6.5 mm diameter, 35 mm length) were inserted into a rigid polyurethane foam block (density of 0.32 g/cm3), a widely used substitute to cadaveric bone with more uniform material properties [17]. The construct was mounted onto the Testometric M350-10 kN test machine. A constant tensile load was applied at a rate of 5 mm/min along the axis of the screw until the screw was released from the test block or broke (Figure 5).

## 3. Results

1. Static axial compression bending test: a total of six CFR-PEEK construct samples, with 6.5 mm pedicle screws and 6.0 mm rods, were tested according to the ASTM F1717 protocol. The test results are summarized in Table 1. The failure mode for all the CFR-PEEK samples tested was rotational slip of the rod-screw link.
2. Fatigue axial compression bending test: a total of six CFR-PEEK construct samples with 6.5 mm pedicle screws and 6.0 mm rods were tested according to the ASTM F1717 protocol. The test results are summarized in Table 2. No failure occurred at increasing load levels up to (and including) 300 N (83% of ultimate axial compression bending load) and the tested samples completed 5 × 10^6^ cycles successfully with no evidence of fractures, loosening of interconnections, plastic deformation of components or the construct, or other signs of failure. Microscopic examination of the components surface using a digital microscope, with a magnification range of x20 to x200 (Keyence VHX-700F series, Keyence Co., IL, USA), did not reveal damage, such as cracks, delamination, or scratches. Construct measurements taken at the completion of 5 × 10^6^ cycles revealed no change in the effective length of the rods (the center-to-center distance between two longitudinally connected locking elements remained as 76 mm), indicating that no permanent bending of the rods occurred. In all constructs, the four connections were intact, with no notable damage or loosening. The distance between the superior surface of the tulip and the rod was measured for all four interconnections in each construct and was found to be within the required range, indicating that no loosening/disassembly of the components occurred during the test. Also, the distances between the UHMWPE blocks at various points were measured for each construct, demonstrating that the blocks remained parallel to each other (i.e., indicating no slippage of the screw heads or bending of the rods has occurred). At a load level of 343 N (95% of ultimate axial compression bending load), the tested specimen failed after 150 cycles by slippage of the polyaxial screw head within the screw tulip.
3. Static torsion test: a total of four CFR-PEEK construct samples, with 6.5 mm pedicle screws and 6.0 mm rods, were evaluated according to the ASTM F1717 standard. The results are presented in Table 3. The failure mode was slippage of the spherical screw head relative to the rod (slippage of the spherical screw head within the tulip).
4. Screw axial pull-out strength test: a total of four samples of CFR-PEEK polyaxial pedicle screws (6.5 mm diameter, 35 mm length) were tested according to ASTM F2193 and ASTM F543 standards as applicable.  Table 4 summarizes the test results. Failure in all the cases involved the polyurethane foam. No failure was detected in any of the screws.

## 4. Discussion

Spine fixation systems made of composite materials such as CFR-PEEK have emerged recently as promising innovations in spine surgery. This study is the first to test the mechanical properties of the CarboClear system, a novel spine fixation system made entirely (screws and rods) of CRF-PEEK composite material.

We tested the CFR-PEEK pedicle screw system using various mechanical tests according to the ASTM standards in order to assess whether its mechanical properties are comparable with published data on other commonly used metal spine fixation systems.

The mean bending yield load and bending ultimate load of the CFR-PEEK system were 322 N and 363 N, respectively, which are comparable to the values reported for the Moss Miami Ti system (DePuy, Warsaw, IN, USA) (299 N and 499 N, respectively) and superior to those reported for the Synergy VLS Open system (Biomet Spine, Broomfield, CO, USA) (214 N and 292 N, respectively) [18]. Bending stiffness of the CarboClear system was greater than the values reported for the titanium systems (CarboClear bending stiffness of 45 N/mm compared to 24 N/m and 33 N/mm for the Moss Miami Ti and the Synergy VLS Open systems, respectively). This is an unexpected and less favorable finding, considering that one of the known advantages of CFR-PEEK implants is their semirigidity compared to metal implants that should enable more load sharing and avoid disadvantages of rigid instrumentation. A higher fraction of carbon fibers dispersed in the PEEK matrix may explain this greater bending stiffness; however, this explanation is unlikely since the CarboClear system is designed to contain approximately 60% of carbon fibers by volume, which should yield stiffness similar to that of bone [11, 12] and not greater compared to metal implants. Further research may be required in order to clarify the nature of this finding.

The CFR-PEEK system showed no signs of fatigue at increasing cyclic axial compression loads up to 300 N (83% of its bending ultimate load) and successfully completed at least 5 × 10^6^ cycles, with no evidence of mechanical failure. This meets the test acceptance criteria, which specifies that all samples tested at loads below 75% of the bending ultimate load should complete 5 × 10^6^ cycles without failure. Both Moss Miami Ti and Synergy VLS Open systems showed inferior fatigue endurance and failed at 75% ultimate load after 0.04 × 10^6^ and 0.98 × 10^6^ cycles, respectively [18]. The superior fatigue endurance of the CFR-PEEK system may be attributed to the higher elasticity of the CFR-PEEK, as well as to the intrinsic properties of the composite material.

The torsional stiffness of the CFR-PEEK system (1.0 Nm/deg) was inferior to the values reported for several titanium systems, e.g., Globus Revolve polyaxial system (Globus Medical, Audubon, USA) (1.3 Nm/deg) [19], USS spine system (DePuy Synthes, Zuchwil, Switzerland) (2.2 Nm/deg) [19], and Moss Miami Ti system (1.8 Nm/deg) [20]. The failure mode of the CFR-PEEK specimens that we tested was a slippage of the spherical screw head relative to the rod (i.e., slippage of the spherical screw within screw tulip). A similar failure pattern, attributed to the coupling mechanism between the polyaxial screw head and its shaft, was found also in the Revolve and USS systems mentioned above [19, 20].

The axial pull-out strength of the CFR-PEEK 6.5 mm pedicle screws observed in our testing (2,037 N) is comparable to the pull-out strength of the Moss Miami 6.9 mm pedicle screw (1,888 N) and the Cotrel-Dubousset 6.5 mm pedicle screw (Medtronic Sofamor Danek, Memphis, TN) (1,895 N) [17]. All screw systems failed at the screw-polyurethane foam interface, with no structural damage to the screw itself.

Our adherence to the ASTM F1717 vertebrectomy model protocol may be considered as a limitation of the study, since the F1717 protocol has been shown to be a worst-case loading condition that may not actually represent loading within activities of daily living [21].

## 5. Conclusion

We demonstrated that the composite material spine fixation system, comprising CFR-PEEK pedicle screws and rods, has mechanical properties comparable to those of other commonly used titanium-made systems, with superior fatigue properties. The fatigue resistance, modulus of elasticity which is very similar to that of bone, radiolucency, and CT/MRI artifact-free feature of spine fixation systems made entirely of CFR-PEEK may offer advantages over traditional spine fixation systems made of metal alloys. Continued clinical research with large patient cohort, variety of surgical indications, and long-term follow-up is required in order to evaluate the performance of the CarboClear system over time.

## Figures and Tables

**Table 1:** Static axial compression bending test results of the CarboClear system.

Sample	Bending yield load (N)	Bending ultimate load (N)	Bending stiffness (N/mm)	Displacement at 2% offset yield (mm)	Elastic displacement (mm)	Bending ultimate displacement (mm)
1	302	312	42	9.5	8.0	11.0
2	310	325	50	7.7	6.5	8.3
3	312	351	48	8.0	6.5	9.5
4	350	401	44	9.4	7.9	11.8
5	325	408	42	9.3	7.8	13.4
6	335	381	46	8.8	7.3	12.2
Mean ± SD	322 ± 18	363 ± 40	45 ± 3.2	8.8 ± 0.8	7.3 ± 0.7	11 ± 1.8

**Table 2:** Fatigue axial compression bending test results of the CarboClear system.

Sample	Load (N)	Percentage of ultimate bending load (%)	No. of cycles to failure	Mode of failure
1	200	55	5 × 10^6^	No failure, test terminated voluntarily
2	210	58	7.7 × 10^6^	No failure, test terminated voluntarily
3	230	63	5 × 10^6^	No failure, test terminated voluntarily
4	277	76	5 × 10^6^	No failure, test terminated voluntarily
5	300	83	6 × 10^6^	No failure, test terminated voluntarily
6	343	95	150	Slippage of the polyaxial screw head within the screw tulip

**Table 3:** Static torsion test results of the CarboClear system.

Sample	Yield torque (Nm)	Torsional stiffness (Nm/deg)	Angular displacement at 2% offset yield (deg)	Elastic angular displacement (deg)
1	12.4	0.9	16.1	14.2
2	10.4	1.1	11.1	9.2
3	12.4	1.2	12.4	10.2
4	15.1	0.9	18.3	16.2
Mean ± SD	12.6 ± 1.9	1.0 ± 0.1	14.4 ± 3.3	12.4 ± 3.3

**Table 4:** Screw axial pull-out test results of CarboClear pedicle screws (6.5 mm diameter, 35 mm length).

Sample	Axial pull-out strength (N)
1	2151
2	1901
3	2041
4	2057
Mean ± SD	2037 ± 103

## Data Availability

The data used to support the findings of this study are available from the corresponding author upon request.
